# A heterogeneous subcontinental mantle under the African–Arabian Plate boundary revealed by boron and radiogenic isotopes

**DOI:** 10.1038/s41598-021-90275-7

**Published:** 2021-05-27

**Authors:** Samuele Agostini, Paolo Di Giuseppe, Piero Manetti, Carlo Doglioni, Sandro Conticelli

**Affiliations:** 1 Istituto di Geoscienze e Georisorse, Sede Principale di Pisa, Consiglio Nazionale delle Ricerche, Via Moruzzi, 1, 56124 Pisa, Italy; 2 Dipartimento di Scienze della Terra, Università degli Studi di Firenze, Via Giorgio La Pira, 4, 50121 Firenze, Italy; 3 Dipartimento di Scienze della Terra, Università La Sapienza, Roma, Piazzale Aldo Moro 5, 00185 Roma, Italy; 4 Istituto Nazionale di Geofisica e Vulcanologia , Via Vigna Murata 605 , 00143 Roma, Italy; 5 Istituto di Geologia Ambientale e Geoingegneria, Sede Principale di Montelibretti, Consiglio Nazionale delle Ricerche, Area della Ricerca di Roma1 - Montelibretti, Via Salaria Km 29, 300, 00015 Monterotondo, RM Italy

**Keywords:** Solid Earth sciences, Geochemistry, Geodynamics, Petrology

## Abstract

The northern and northwestern margins of the Arabian Plate are a locus of a diffuse and long-lasting (early Miocene to Pleistocene) Na-alkali basaltic volcanism, sourced in the asthenosphere mantle. The upwelling asthenosphere at the Africa–Arabia margin produces very limited magma volumes in the axial zone. Therefore, portions of hot, fertile mantle continue their eastward migration and are stored at shallower depths under the 100-km thick Arabian lithosphere, which is much thinner than the African one (≈175 km): this causes the occurrence and 20-Ma persistence of magma supply under the study area. Erupted basalts sampled a continuous variation of the mantle source, with a striking correlation among temperature, pressure and isotopic composition shifting between two end members: a 100 km-deep, more depleted source, and a 60 km-deep, more enriched one. In particular, we observed an unusual variation in boron isotopes, which in the oceanic domain does not vary between more depleted and more enriched mantle sources. This study shows that, at least in the considered region, subcontinental mantle is more heterogeneous than the suboceanic one, and able to record for very long times recycling of shallow material.

## Introduction

The geochemical and isotopic signature of basaltic magmas can provide unique insights into the characteristics of the upper mantle and of its dynamics, with fundamental implications for the tectonic framework. Alkaline basaltic magmas are mostly found in the ocean islands and in within-plate continental settings, as well as continental rifts. Diffuse alkali basaltic magmatism is also in some cases associated with strike-slip and transform faults system, and strike-slip related transtensional tectonic regimes, where lithospheric thinning and/or tearing may lead to magma formation through decompression melting of mantle with sub-lithospheric and/or lithospheric geochemical characters, or a combination of both^1^.

Unlike magmas generated in ocean islands and continental rifts, mantle-derived partial melts erupted in a within-plate continental tectonic setting, as well as those along continental strike-slip areas, may interact with the lithospheric mantle and/or the crust during their ascent to the surface. For this reason, the primary character of intraplate-type basaltic magmas ascending through the continental lithosphere may be significantly modified by fractional crystallisation, eventually matched by crustal assimilation. Several models regarding the structure and geochemical evolution of the Earth’s mantle suggest that over billions of years, ancient subducted components continuously return surface materials to depth, where they persist as metasomatic domains in the deep mantle to form a highly heterogeneous mantle^2^.

Continental intraplate-type basalts exhibit a wide range of chemical and isotopic compositions: (i) some are derived from a depleted mantle source and retain the characteristics of a pristine asthenosphere, whereas (ii) others display signatures typical of continental crust or metasomatically enriched domains, reflecting some contributions from the continental lithosphere in the source^3,4^, or during magma ascent. Basaltic products erupted in these contexts may be characterised by variable Large Ion Lithophile Element (LILE), Light Rare Earth Element (LREE), High Field Strength Element (HFSE) and radiogenic (Sr–Nd-Pb) and stable (e.g., B) isotopic compositions^5^.

Stable and radiogenic isotopes are extremely useful as tracers of long-lasting heterogeneities in the Earth’s mantle. In particular, boron (B) represents an ideal tracer of the chemical evolution of the mantle. Because boron is both incompatible in the mantle and highly mobile in aqueous fluids, the injection of shallow materials in the mantle strongly impacts mantle B concentrations and isotopic signatures^6,7^. Boron isotope estimates for MORBs, OIBs and the mantle have been recently reviewed^8^: fresh unaltered MORBs were found to vary within a narrow range (δ^11^B = − 6 to − 8‰) when not affected by assimilation of crustal material, whereas OIBs from the “uncontaminated” mantle show values that vary from δ^11^B = − 3 to − 12‰, differing less than 4–5‰ from MORBs. Few data are available on Na-alkali basalts emplaced on the Continental Lithosphere, which are mostly sourced in the sub-lithospheric mantle^9^, as it is for OIBs. However, the alkali basalts of Western Anatolia, less than 2000 km from our study area, with a radiogenic isotope signature typical of an “uncontaminated” mantle, have higher δ^11^B values (≈− 3 to 0‰ both for Biga Peninsula and Kula)^10^.

**Figure 1:**
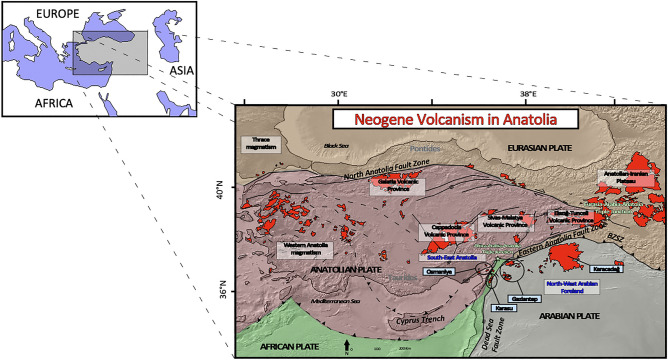
Simplified tectonic and volcanological map of the region where Eurasian, Anatolian, African and Arabian plates meet. Green stars are the African-Arabian-Anatolian and Anatolian-Arabian-Eurasian triple junction, respectively. Neogene volcanic activity is in red; study areas are highlighted. DEM created with GeoMapApp version 3.6.8 (http://www.geomapapp.org).

Here, we present the results of a comparative geochemical and isotopic study on Miocene-Holocene basaltic magmas erupted along the northwestern margin of the Arabian Plate, close to the Africa–Arabia–Anatolia triple junction (Osmaniye and Karasu sectors) and in the northwestern Arabian foreland (Gaziantep Basin and Karacadağ shield volcano). The geodynamic evolution of the Eastern Mediterranean Region was dominated by the northward motion of the African and Arabian Plates, which converged towards the Eurasian Plate starting in the Late Cretaceous^11^. While in the north this convergence induced the Neotethys Ocean to subduct along the Eurasian margin, farther south the counter-clockwise rotation of the Arabian Plate at the beginning of the late Oligocene to early Miocene led to the initial opening of the Gulf of Aden, followed by the development of the Red Sea and Gulf of Suez Rift Systems, so that the African Plate and the Arabian Plate began to separate^12^. In response to these movements, the Dead Sea Fault developed as a left-lateral strike slip fault, transferring the opening of the Red Sea Rift to the north, and finally separating the Arabian Plate from the African one. The Arabia–Eurasia convergence culminated at 13 Ma (middle Miocene) with collision along the Bitlis-Zagros Suture Zone (BZSZ^13^; Fig. 1). This impact gave rise to shortening and uplift in the East Anatolia region (e.g. Anatolian-Iranian Plateau and Central Anatolian Plateau), the formation of the middle Miocene North Anatolia Fault Zone (NAFZ) and the Pliocene strike-slip East Anatolia Fault Zone (EAFZ)^14^. In this tectonic scenario, Arabia, Africa and Anatolia meet at the Maras Triple Junction, the point in which the Dead Sea Fault Zone (DSFZ), the EAFZ and the north-eastern termination of the Cyprus trench (Fig. 1) all meet up. Extensive intraplate-like volcanism occurred in South-East Anatolia and the north foreland of the Arabian Plate (Fig. 1).

West of the Arabian Plate, several tectonic basins with volcanic fields developed near the Maras Triple Junction, in which the African, Arabian, and Anatolian Plates meet (Pazarcık-Narlı, early Miocene)^15^. Strike-slip related volcanic activity also occurred during the Pliocene–Pleistocene along the northern termination of the DSFZ, in the Karataş-Osmaniye fault zones (2.3–0.12) Ma^15,16^ and in the Karasu Basin (1.6–0.06 Ma)^17–19^. In the northern part of the Arabian Plate, intracontinental basaltic volcanic activity was dominant throughout the Miocene within the Gaziantep Basin (21.2–7.0)^20,21^ and occurred in three different pulses in the Karacadağ volcanic complex^22^. The first pulse (Siverek Stage) started during the late Miocene to early Pliocene (11.0–2.7 Ma)^23^. Scattered lava flows ascribed to this first stage also crop out around Urfa, spanning in age from 12.1 Ma to 6.65 Ma. The second volcanic phase (Karacadağ Stage) developed during the Pleistocene (1.9–1.0 Ma), producing the main edifice of the volcanic complex. The youngest products, belonging to the Ovabağ Stage emplaced during the Pleistocene-Holocene (0.4–0.01 Ma), crop out only in the eastern and south-eastern sectors of the volcano.

## Results

**Figure 2:**
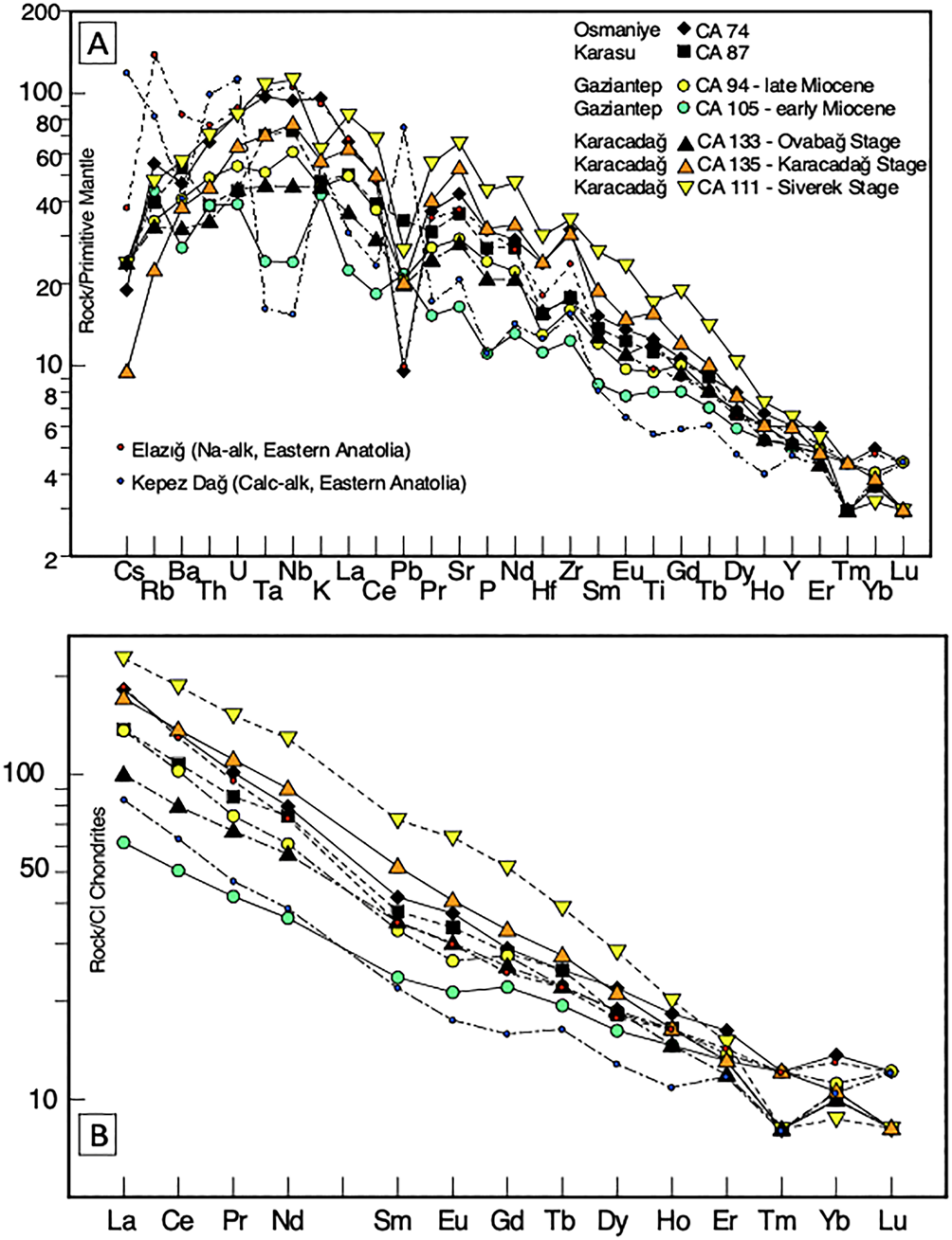
Distribution of incompatible trace element and Rare Earth Element (REE) of selected alkali basalts. (**A**) Primitive Mantle-normalised diagrams for selected samples. (**B**) Chondrite-normalised REE diagrams for the same samples. Na-alkaline basalt from Elazığ in Eastern Anatolia^25^ and a subduction-related basaltic andesite from Kepez Dağ volcanic complex in Central-Eastern Anatolia^24^ are reported for comparative purposes. Primitive Mantle and Chondrite normalisation factors from McDonough and Sun^26^.

We selected 18 representative basanites, basalts and basaltic andesites among a complete set of one hundred samples from the African-Arabian plate boundary (Fig. 1). The most relevant data are reported in Table 1 (full chemical data in Supplementary Table 1, whilst available ages are reported in Supplementary Table 2). Samples display a narrow SiO_2_ range (41.4–52.3 wt.%) and a wide MgO range (6.5–12.1 wt.%) and have a Na-alkaline affinity. Their Primitive Mantle-normalised multi-element patterns are characterised by a hump with a maximum at Nb–Ta, typical of within plate-type magmas (Fig. 2A). Differentiated magma compositions, and all of the early Miocene samples from Gaziantep, retain some LILE enrichment relative to HFSE and a small negative Nb–Ta anomaly (CA 105, Fig. 2 and see also Supplementary Fig. 1). However, these patterns are quite different from typical arc magmas (e.g., the basaltic andesites of similar age cropping out at Kepez Dağ, Eastern Anatolia)^24^, as they are less enriched in fluid mobile elements (e.g. Cs, Rb, Th, Ba) and lack a positive Pb-spike and negative Ti anomaly. REE element patterns for Osmaniye, Karasu and Gaziantep samples (Fig. 2B) are characterised by negative trends, with steeper slopes on the LREE side, accompanied by minor but consistent HREE fractionation, in particular for Karacadağ samples.

**Table 1:** Selected Major Elements (wt%), Trace Element Ratios, and Isotope Compositions of studied samples.

Sample	CA 74	CA 76	CA 78	CA 92	CA 81	CA 87	CA 89	CA 94	CA 99	CA 104	CA 105	CA 111	CA 112	CA 121	CA 123	CA 126	CA 135	CA 133
Unit	Osm	Osm	Osm	Osm	Karasu	Karasu	Karasu	Gaz (LM)	Gaz (EM)	Gaz (EM)	Gaz (EM)	KD (Siv)	KD (Siv)	KD (Siv)	KD (KD)	KD (KD)	KD (KD)	KD (OB)
Rock Type	*Basanite*	*Basalt*	*Basalt*	*Basalt*	*Basalt*	*Tr Bas*	*Basalt*	*Basalt*	*Bas And*	*Bas And*	*Bas And*	*Basanite*	*Basalt*	*Basalt*	*Basalt*	*Basalt*	*Basanite*	*Basalt*
SiO_2_	45.54	46.02	47.93	48.15	50.77	46.78	46.59	46.84	50.94	52.19	52.30	41.40	50.33	46.82	45.51	47.00	43.94	47.60
TiO_2_	2.47	2.40	1.78	1.87	1.69	2.26	2.18	1.89	1.53	1.49	1.60	3.33	2.14	2.30	2.35	2.33	3.10	2.42
MgO	7.72	8.70	8.70	8.42	6.50	9.05	9.96	9.01	7.71	7.70	8.54	9.81	6.63	9.27	12.12	9.72	10.11	9.46
Na_2_O	4.97	3.27	3.12	3.14	3.43	3.53	3.42	3.53	3.09	3.09	3.14	4.15	3.20	3.34	2.73	2.82	3.54	3.31
K_2_O	2.72	1.15	0.68	0.72	0.86	1.37	1.12	1.29	0.72	0.89	1.21	1.75	1.03	1.18	1.02	1.02	1.62	1.29
*Mg#*	*70.22*	*66.95*	*67.80*	*65.21*	*60.21*	*69.74*	*66.35*	*68.00*	*66.94*	*69.52*	*69.46*	*74.02*	*60.77*	*68.81*	*71.31*	*66.98*	*69.25*	*68.94*
Sr/Y	32.8	30.9	27.7	27.2	16.1	33.1	29.7	26.0	13.1	12.3	15.0	46.9	17.2	23.5	29.2	30.0	41.4	25.0
Th/Nb	0.086	0.088	0.193	0.122	0.196	0.064	0.067	0.097	0.142	0.173	0.195	0.075	0.141	0.103	0.076	0.080	0.071	0.090
Nb/U	36.4	45.6	27.1	43.2	19.6	53.4	53.2	36.5	21.2	17.3	19.9	43.6	24.3	34.6	52.6	55.0	39.2	33.4
Rb/Sr	0.0390	0.0142	0.0144	0.0111	0.0455	0.0331	0.0302	0.0350	0.0587	0.1034	0.0803	0.0218	0.0370	0.0354	0.0219	0.0127	0.0127	0.0347
B (µg*g^−1^)	4.8					2.5		2.5	4.0		3.0	2.5		1.9	1.1		2.0	2.9
δ^11^B (‰)	− 2.30 ± 0.13					− 5.60 ± 0.13		− 7.60 ± 0.22	− 1.69 ± 0.11		− 3.13 ± 0.15	− 3.50 ± 0.17		− 5.84 ± 0.19	− 5.23 ± 0.08		− 4.77 ± 0.24	− 6.65 ± 0.07
(^87^Sr/^86^Sr)_i_	0.70301	0.70339	0.70404	0.70390	0.70464	0.70343	0.70343	0.70365	0.70486	0.70506	0.70500	0.70337	0.70447	0.70400	0.70389	0.70354	0.70317	0.70376
(^143^Nd/^144^Nd)_i_	0.51296	0.51290	0.51279	0.51289	0.51269	0.51280	0.51290	0.51282	0.51268	0.51266	0.51267	0.51269	0.51271	0.51282	0.51282	0.51304	0.51293	0.51283
(^206^Pb/^204^Pb)_i_	19.059	18.957	19.043	19.006	18.886	18.114	19.204	19.025	18.962	19.221	19.027	18.911	18.796	18.953	18.977	19.139	18.993	19.131
(^207^Pb/^204^Pb)_i_	15.599	15.625	15.699	15.665	15.704	15.558	15.649	15.672	15.705	15.740	15.736	15.589	15.689	15.669	15.668	15.622	15.670	15.611
(^208^Pb/^204^Pb)_i_	38.812	38.822	39.165	38.996	39.117	37.930	39.033	39.110	38.979	39.191	39.325	38.770	39.063	39.022	39.018	38.871	38.912	38.839

*Osm* Osmaniye, *Gaz* Gaziantep, LM Late Miocene, EM Early Miocene, KD Karacadağ, Siv Siverek, OV Ovabağ, *Tr Bas* Trachybasalt, *Bas And* Basaltic Andesite.

For δ^11^B (‰) values the 2σ in− run error is also reported.

Full geochemical data available in Supplementary Table 1.

### Radiogenic isotopes

**Figure 3:**
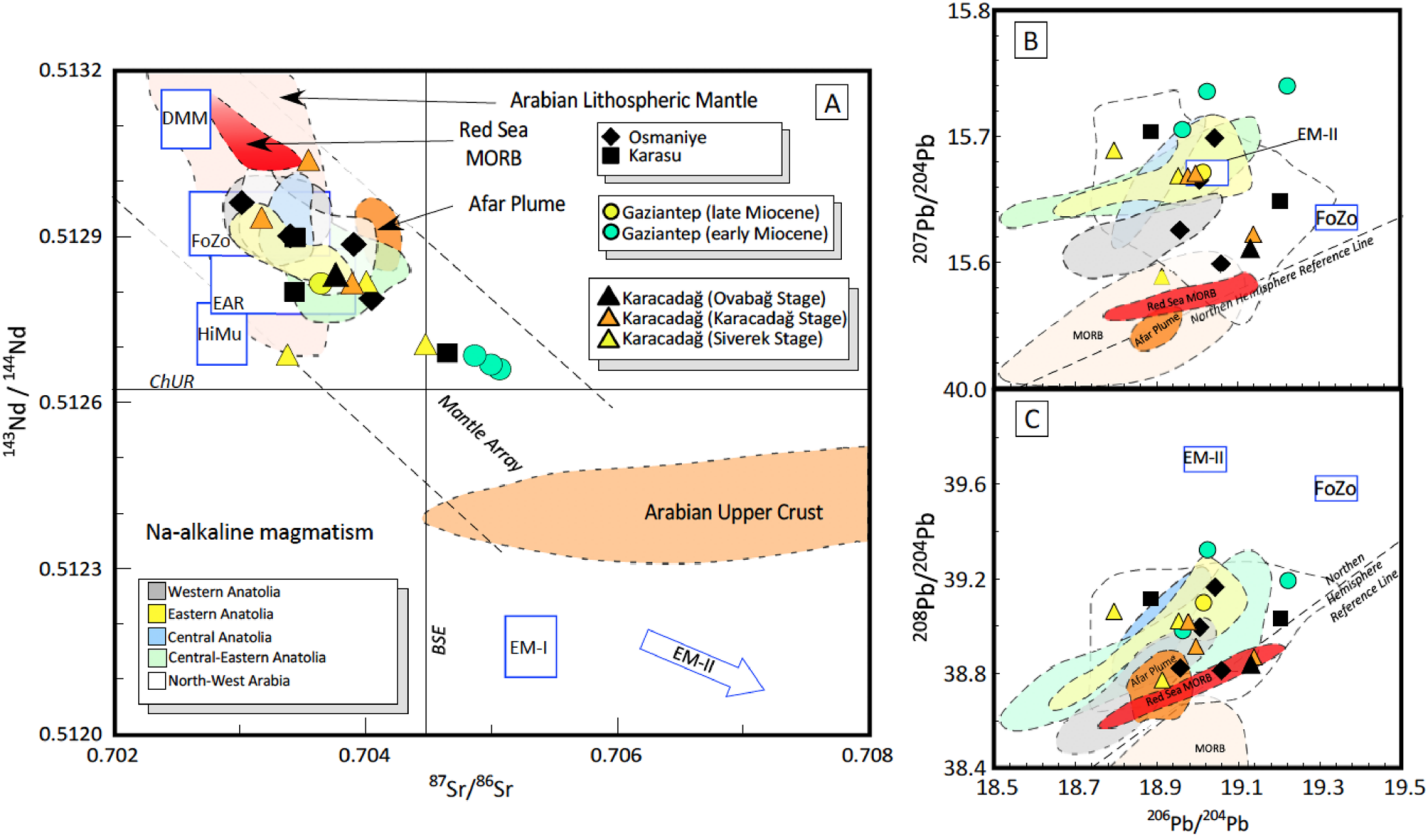
Sr–Nd-Pb radiogenic isotope compositions of Neogene volcanic rocks around Anatolia-Africa-Arabia plate borders. (**A**) ^143^Nd/^144^Nd *vs.* ^87^Sr/^86^Sr isotopic ratios for the studied rocks. BSE is the Bulk Silicate Earth; ChUR is the Chondritic Uniform Reservoir.; (**B**,**C**) ^207^Pb/^204^Pb *vs.* ^206^Pb/^204^Pb and ^208^Pb/^204^Pb *vs.* ^206^Pb/^204^Pb diagrams. Dashed line indicates the Northern Hemisphere Reference Line (NHRL). Mantle end-members: DMM (Depleted MORB Mantle); FoZo (Focal Zone), HiMu = High µ (µ = ^238^U/^204^Pb ratio), EM-I (Enriched Mantle I), and EM-II (Enriched Mantle II). EAR = European Asthenospheric Reservoir. NHRL and fields of Mantle end-embers same as Agostini et al.^27^. Arabian Upper Crust, Arabian Lithospheric Mantle, Red Sea MORB, and Afar plume as in Ma et al.^28^. Fields showing the isotopic composition of Na-alkaline volcanic rocks are from: Kula (Western Anatolia)^29^, Cappadocia (Central Anatolia)^30^, Sivas, Kangal and Arguvan (Central-Eastern Anatolia)^24^; Elazığ (Eastern Anatolia)^25^; Al Ghab, Homs, Aleppo, Shin Plateau and Harrat Ash Shaam (Arabian Plate)^28,31^.

Sr–Nd-Pb radiogenic isotopes are reported in Table 1 and plotted in Fig. 3 (full isotope data in Supplementary Table 2). Studied samples are characterised by a range of variation spanning from 0.70301 to 0.70506 and from 0.51304 to 0.51266 for ^87^Sr/^86^Sr_(i)_ and ^143^Nd/^144^Nd_(i)_, respectively. The studied rocks mostly fall in the depleted mantle quadrant of the ^87^Sr/^86^Sr_(i)_ *vs.* ^143^Nd/^144^Nd_(i)_ diagram, within the so-called Mantle Array. However, while Nd isotope values are always above the ChUR (Chondritic Uniform Reservoir) value, some ^87^Sr/^86^Sr values fall to the right of the BSE (Bulk Silicate Earth) line and some samples deviate from the mantle array, shifted toward more radiogenic Sr. The latter include most Miocene samples, as all of the basaltic andesites from Gaziantep, the sample CA 112 from the Siverek Stage of Karacadağ, and one Pleistocene samples, CA 81 from Karasu volcanism: interestingly, these are also the most evolved samples of each subset and, as indicated above, the ones retaining a negative Ta-Nb anomaly in the spider diagrams (Supplementary Fig. 1).

In contrast, most of the rocks emplaced during the Pleistocene-Holocene (Osmaniye, Karasu and Ovabağ Stage of Karacadağ) fall within the FoZo-EAR fields, showing values typical of Na-alkaline rocks emplaced throughout Western (e.g., Kula and Biga Peninsula)^29^, Central and Eastern Anatolia (Kızılırmak-Acıgöl, Nevşehir, Sivas, Kangal, Arguvan, Elazığ and Karakoçan)^24^, as well as in the Arabian Plate (e.g., Aleppo, Shin Plateau and Harrat Ash Shaam)^28^. This suggests that these rocks derive from a common depleted mantle source (Fig. 3A), located in the mantle asthenosphere. In contrast to the Sr–Nd isotopes, the Pb isotope contents in studied rocks show considerable scatter. In particular, the ^206^Pb/^204^Pb_(i)_ ratio ranges from 18.80 to 19.22, the ^207^Pb/^204^Pb_(i)_ ratio from 15.59 to 15.73, and ^208^Pb/^204^Pb_(i)_ from 38.77 to 39.32, depicting rough positive trends in both diagrams shown in Fig. 3B,C. With respect to the surrounding mantle-derived basalts, the ^207^Pb/^204^Pb_(i)_ ratios of the studied rocks is similar to those of other alkali basalts from the Anatolia region, and slightly higher than depleted mantle compositions such as MORB, Red Sea MORB and Afar.

### Boron isotopes

**Figure 4:**
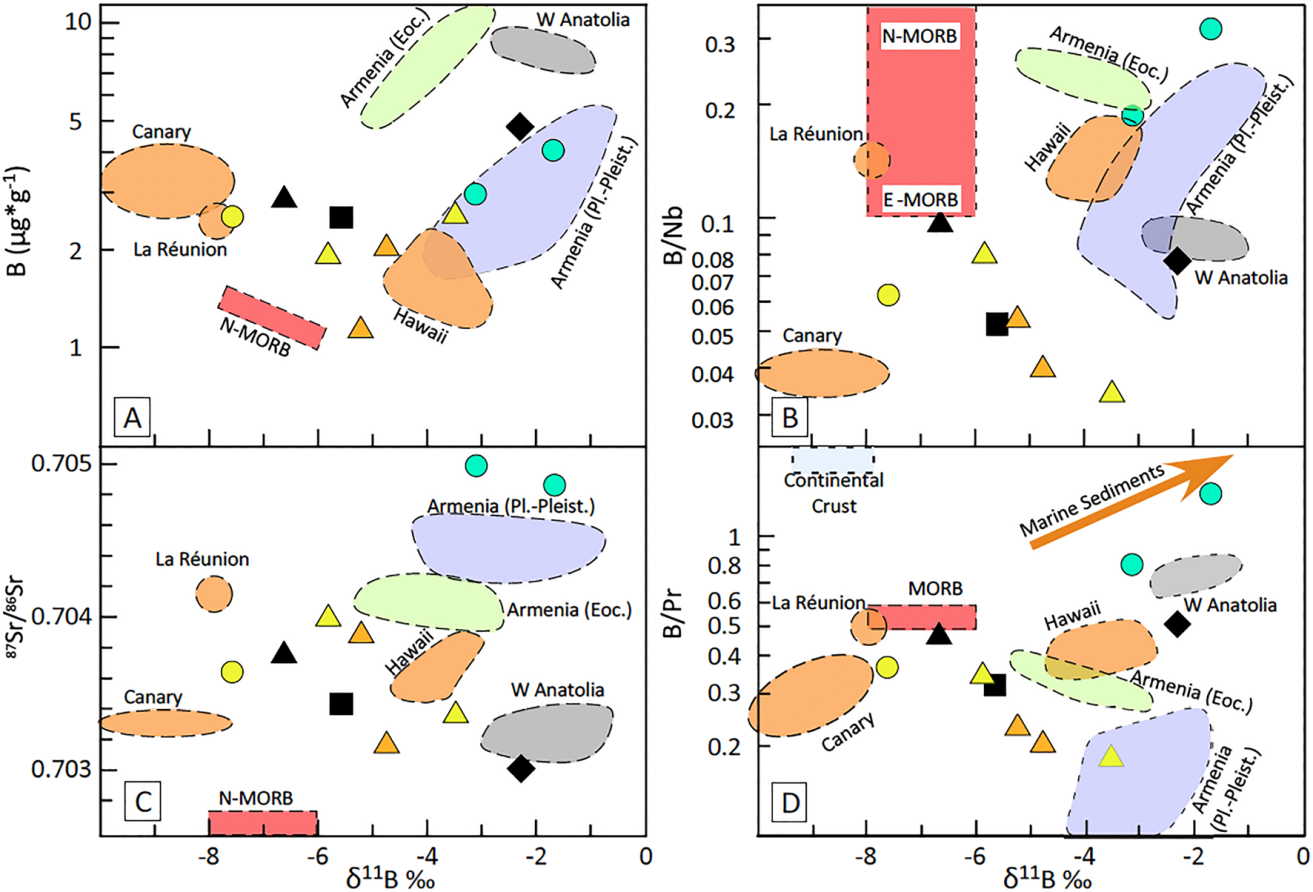
Boron isotopes ratios of studied rocks with respect to some continental and oceanic basalts. (**A**) δ^11^B *vs.* B contents; (**B**) δ^11^B *vs.* B/Nb; (**C**) δ^11^B *vs.* ^87^Sr/^86^Sr; (**D**) δ^11^B *vs.* B/Pr. Variation field of MORBs^32^, OIBs^33^ and collision-related Armenian rocks^35^ are also shown for comparative purposes. Symbols as in Fig. 3.

We selected ten representative sample of each studied location: most of them are primitive with small or negligible degree of differentiation, only two samples of Gaziantep show some degree of evolution. The studied samples (Table 1, Fig. 4) are characterised by relatively low boron concentrations (1–5 µg/g) and B/Nb ratios, as well as negative B isotope compositions ranging from − 7.6 to − 1.7 ‰ (Fig. 4A). Most of the studied samples define a negative correlation in both δ^11^B *vs.* B contents (Fig. 4A), δ^11^B *vs.* B/Nb or B/Pr (Fig. 4B,D) and δ^11^B *vs.* ^87^Sr/^86^Sr_(i)_ (Fig. 4C) diagrams, with the exception of two evolved samples from the early Miocene phase of Gaziantep activity. Boron concentrations are higher than MORBs and similar to some representative OIBs (Hawaii, La Réunion and Canary, Fig. 4A), whereas B/Nb and B/Pr ratios are lower than those observed for N-MORBs (Fig. 4B) and δ^11^B values vary from typical MORB and OIB values to higher value spanning the range from Primitive Mantle to Western Anatolia alkali basalt values.

## Discussion

Volcanic activity in the region started in the early Miocene (21 Ma) with the emplacement of abundant Na-alkaline products and subordinate silica-oversaturated basaltic and andesitic rocks in the north sector of the Arabian foreland, within the Gaziantep Basin. In the middle-late Miocene a new pulse of magmatism occurred within this basin, while Siverek Stage Na-alkaline volcanism took place in western and southern Karacadağ, namely around the cities of Urfa, Siverek and Viranşehir; as for the early Miocene lavas, some subordinate silica-oversaturated basalts are found in the Siverek Stage products^23^. Subsequently, at the beginning of the Pliocene through to the Pleistocene-Holocene, two volcanic phases (Karacadağ and Ovabağ Stages) characterised Karacadağ volcanic activity, with the production of abundant Na-alkaline magmas. Lastly, during the Pleistocene to recent, strike-slip related magmatism with Na-alkaline affinity developed along the northern sector of the Dead Sea Fault Zone (e.g., Karasu Valley) and a few km to the north, along the Karatas-Osmaniye strike slip fault (Osmaniye area) in the Eastern Anatolia Fault Zone. Note that even if the study samples come from different areas and cover a time span of more than 20 Ma, they have practically the same petrography and major element geochemistry.

**Figure 5:**
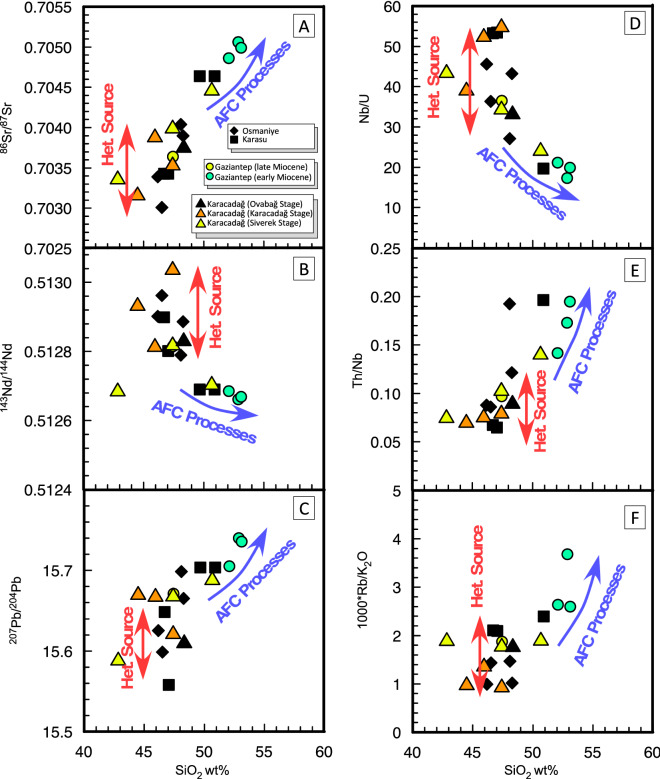
Variations in radiogenic isotope and trace element ratios due to mantle heterogeneity *or* crustal contamination. (**A**–**C**) ^87^Sr/^86^Sr, ^143^Nd/^144^Nd, and ^207^Pb/^204^Pb *vs.* SiO_2_**; **(**D**–**F**) Nb/U, Th/Nb, and 1000*Rb/K_2_O *vs.* SiO_2_ diagrams. Red arrows indicate variation due to source heterogeneity, blue arrows indicate variation due to open-system magma evolution (i.e. assimilation plus crystal fractionation processes).

The occurrence of large trace element and isotopic variations led us to investigate the processes responsible for these trends. In particular MgO (6.5–12.1 wt.%) and Mg# (60–74) variations indicate that some of the studied rocks are not in equilibrium with the mantle source, and that evolutionary processes involving fractional crystallisation (FC) and/or crustal assimilation (AFC, Assimilation plus Fractional Crystallisation) played a significant role. When observing B isotopes (Fig. 4) it is immediate to note that some samples with higher δ^11^B values fall outside from main trends and retain higher ^87^Sr/^86^Sr, B/Nb and B/Pr ratios. Boron and Praseodymium have close incompatibility in the mantle, and very similar behaviour in melts, but B is enriched with respect to Pr in the crust, and it is much more mobile in fluids. As a consequence, samples with higher B/Pr ratios (e.g. B/Pr > 0.7, Fig. 4D) may have undergone significant amounts of crustal digestion. To assess the role of FC and in particular of AFC processes, we plotted in Fig. 5 the SiO_2_ content of studied rocks *vs.* radiogenic isotope ratios and selected trace element ratios. From Fig. 5 it is evident that, both when considering Sr, Nd and Pb isotopes or Nb/U, Th/Nb or K/Rb ratios we have a certain degree of correlation with SiO_2_. In particular some ratios, such as Nb/U (17–55) and Th/Nb (0.064–0.196) span a range from values typical of MORBs and OiB-HiMu lavas (Nb/U > 40 and Th/Nb < 0.1) in the most primitive samples, to typical continental crust values in the relatively more evolved samples (Fig. 5D,E), and more evolved samples show lower ^143^Nd/^144^Nd, higher ^207^Pb/^204^Pb, and more importantly ^87^Sr/^86^Sr > 0.7045, that is the value of Bulk Silicate Earth.

These characteristics imply digestion of small amounts of crustal material during magma ascent, in some samples, especially those with SiO_2_ > 50 wt%. In contrast, there is an evident vertical (Fig. 5A–D) or scattered (Fig. 5E,F) distribution for samples with SiO_2_ < 50%. As an example, significant Sr–Nd isotope variations are observed in the less evolved samples (e.g., ^87^Sr/^86^Sr varying from 0.70312–0.70404 for SiO_2_ < 48%, Fig. 5A). This implies that we can rule out the occurrence of significant amount of crustal contamination for samples with SiO_2_ < 50%, and attribute their isotope variation to the occurrence of a heterogeneous source ranging widely from depleted N-MORB type mantle to Primitive Mantle values. Moreover, the negative correlation observed in Fig. 4C between B isotopes and B/Pr ratios, with a trend shifting from typical mantle values towards the bottom right of the diagram, clearly point out the occurrence of a source heterogeneity. Indeed, this trend can not be explained with any contamination process, because continental crust digestion will shift values toward the upper left and marine sediment toward the upper right. In addition, large variations occurring in major and trace element of most primitive samples, also suggest that melting depth and melting degree may significantly vary among and within the various suites. Then, in the following paragraphs, all the samples with SiO_2_ > 50% will be no longer considered.

**Figure 6:**
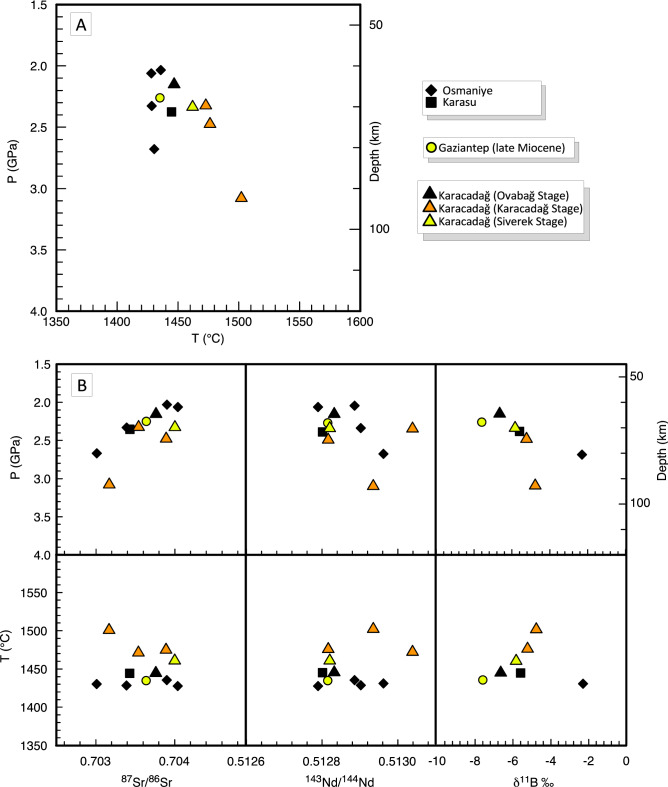
Covariations of intensive parameters and isotope compositions for samples with SiO_2_ wt% < 50. (**A**) Temperatures *vs.* pressures of magma segregation calculated for selected primitive basaltic rocks. (**B**) P and T *vs.* ^87^Sr/^86^Sr, ^143^Nd/^144^Nd, and δ^11^B diagrams.

The mantle segregation temperature and pressure of primary melts were estimated using their major element compositions, as described in detail in the Supplementary Materials. Results (Fig. 6) show that segregation temperatures (T °C) vary from 1428 to 1501 °C, whereas estimated pressures (P) vary from 2.03 to 3.06 GPa, corresponding to a depth of ≈61–93 km, excluding anomalously higher values for sample CA 111 (Supplementary Table 3). These results are in good agreement with other P–T estimates reported in recent literature (see Supplementary Materials for more details). Thus, magma segregation occurred mostly in the garnet stability field, or in the garnet-spinel transition zone^36^. Interestingly, segregation temperatures and pressures are quite well correlated with both radiogenic and B isotope ratios. In particular a well-defined negative trend is observed for pressure and ^87^Sr/^86^Sr, with the younger magmas characterised by the highest pressures and the lowest Sr-isotopic compositions (Fig. 6B). These trends indicate a heterogeneous mantle source, with a less depleted domain towards the surface and a more depleted component at greater depths.

Variations in radiogenic isotopes are the most distinctive features of a mantle source shifting from Primitive to Depleted Mantle domains, and the Sr–Nd variations trends depicted by our samples are therefore quite common. However, radiogenic isotopes, B isotopes and T-P estimates together highlight two peculiarities: (i) although more depleted sources are usually shallower than those more akin to a Primitive Mantle, here depletion increases with depth; (ii) in theory, B isotopes should not vary significantly between Primitive and Depleted Mantle values. As B isotope fractionation at magmatic temperatures is negligible, magma fractionation responsible for depletion of the mantle can affect B contents but should not change the B isotope signature.

The B isotope composition of the pristine mantle has not been directly measured: because the pristine mantle has very low B concentration, mantle peridotites have a very low B content. Moreover, the high mobility of B in fluids causes a significant shift of B isotopic values in mantle rocks with either a minimal metasomatic imprint or even a very low degree of alteration. The best estimate for the B isotope composition of the pristine mantle comes from MORBs or OIB sourced in the uncontaminated mantle^8^. The δ^11^B values of MORBs were recently measured accurately in both fresh unaltered MORBs sourced from Depleted Mantle^32^ and some OIBs sourced from uncontaminated mantle akin to Primitive Mantle^34^. Fresh MORBs vary in a narrow interval, δ^11^B = − 7 ± 1‰, and most OIBs fall in a similar range, δ^11^B = − 7 ± 3 ‰. This indicates that the mantle is relatively homogeneous in terms of the B stable isotope composition, unless it is modified by recycling of subducted material. In facts, up to 6% variation of δ^11^B ratio (from − 20‰ to + 40‰) can be found in shallow environments, from continental sediments to seawater, from dehydrated exhumed terranes to seafloor serpentinites, and a wide range of values is recorded by arc lavas worldwide (− 15‰ to + 10‰)^7,8,37^. Hawaiian rocks have slightly heavier values (δ^11^B = − 5 to − 3 ‰)^33^ than most OIBs. In contrast, OIB-type intraplate Na-alkali basalts from Western Anatolia, sourced in the mantle under the continental lithosphere, retain higher values (− 3 to − 1‰)^10^. The South-East Anatolian basalts, even excluding the two most evolved samples affected by AFC processes (i.e., the two early Miocene samples from Gaziantep, Fig. 4), display a continuous trend of variation from typical MORB-OIB values to values like those of Western Anatolian basalts (− 2 to − 8‰). In addition, although oceanic basalts show no correlation between B isotopes and radiogenic isotopes (Fig. 4C), B isotope variations in South-East Anatolia primitive basalts are fairly well correlated with Sr isotopes, B/Nb and B/Pr ratios (Fig. 4B,D) as well as temperature and pressure estimates (Fig. 6). We can conclude that the subcontinental asthenospheric mantle, at least the one present under the Anatolia and Arabia regions, is heterogeneous even when considering B stable isotopes, and that there is a somewhat continuous shift between a Réunion-like more enriched source and a more depleted source (Fig. 4C), which is higher in δ^11^B, but B-depleted with respect to highly incompatible elements (e.g. Nb, Fig. 4C) as well as elements with same mantle incompatibility as LREEs (e.g., Pr, Fig. 4D). To what extent this mantle can be considered pristine and to what degree these variations are due to recycling of old (or very old) crustal and lithospheric material is still a matter of debate. However, recycling of shallow material in the deep mantle should produce an increase in B concentrations, whereas South-East Anatolian basalts display low B contents (Fig. 4A), and lower B/Pr ratios, even in some of the samples retaining higher B isotopic signature. The B isotope composition of some of these samples is similar to that of Armenian volcanism, sourced from a metasomatised mantle. However, these latter have higher B/Nb, a positive correlation of B/Nb *vs.* δ^11^B (Fig. 4B), higher ^87^Sr/^86^Sr ratios, and an almost flat ^87^Sr/^86^Sr *vs.* δ^11^B trend (Fig. 4C). These features were interpreted as related with the occurrence of significant amount of amphibole in the mantle source^35^.

Previous studies on volcanism in the North Arabian Plate hypothesized the presence of hydrous phases in the mantle source, such as amphibole and/or phlogopite-rich veins, or a combination of the two, as also supported by the occurrence of metasomatised lithospheric mantle xenoliths and amphibole-rich cumulate crustal xenoliths^38,39^. Studied samples are characterised by a pronounced negative Pb anomaly in the Primitive Mantle-normalised trace elements (Fig. 2), which may indicate the presence of residual amphibole and/or phlogopite in the source, given that both amphibole or phlogopite are considered to represent a main repository for Pb in the mantle^39^. Thus, hydrous phases may occur in the mantle source of the studied rocks; it follows that this mantle was, at some time, probably enriched by recycling of crustal and lithospheric material. The occurrence of amphibole in the mantle source paragenesis of the studied rocks should also significantly affect their B isotope composition, given that amphibole is a main B repository in the mantle and can retain B for a very long time^35^.

### Magmatism and geodynamics

Volcanism in the study area is quite peculiar because it results from a number of distinct magmatic pulses showing similar geochemical characters, emplaced in a relatively limited area, over a very long period of more than 20 Ma, and in varied tectonic settings, e.g., in close proximity to continental transform fault systems, close to the Arabia-Africa-Anatolia triple junction, or in the foreland of the Arabia-Eurasia convergence. These lavas, despite their wide age span (> 20 Ma) and their different tectonic location, share similar petrographic and geochemical characteristics and limited and continuous variations in their mantle source between a more-enriched (Réunion-like) and a more depleted (West Anatolian-like) end members. This rather unusual, peculiar feature of this magmatism is also found in other older alkali basalts clustering close to the western margin of the Arabian Plate, from southeastern Turkey to Yemen and several authors noted that all these magmas are characterised by similar geochemical and isotopic compositions resembling the typical Afar fingerprint, and suggested that volcanism has with time migrated northward from the Afar Plume to the Arabian Plate. For some authors^40^, this migration is linked to the flattening of the Afar plume head and its northward channelling below Arabia. In contrast, Shaw et al.^41^ stated that the magmas emplaced in the Harrat Ash Shaam (Jordan) were geochemically and isotopically akin to the late Cenozoic volcanism throughout the Arabian Plate, but quite different from magmas emplaced in the south-western portion of the Arabian Plate (Yemen), and that only the latter were eventually affected by the Afar plume. Pliocene to Pleistocene magmatism of the Harrat Ash Shaam has been tentatively ascribed to heating of the base of the lithosphere by an anomalously hot sublithospheric mantle^42^, whereas Sobolev et al.^43^ proposed a direct relationship between the Dead Sea Fault Zone-Red Sea Rift system and the Afar plume to explain igneous activity along the fault zone. These authors explained the asymmetry and the difference in topography between the western and eastern sides of the Red Sea and Dead Sea Fault with the presence of a hot mantle adjacent to the eastern shoulder of the rift and a cold lithosphere beneath the western side, with the boundary of the thermal asthenosphere below the African Plate deeper than the one beneath the Arabian Plate. Doglioni et al.^44,45^ noticed that, in general, the eastern side of continental rift zones is usually more elevated than the western one. Since the mantle becomes depleted in Fe after melting, it moves “eastward” relative to the lithosphere: the higher topography of the eastern side of the rift is therefore interpreted in terms of isostatic adjustment (lower thermal subsidence). Due to the net rotation of the lithosphere, the depleted and lighter sub-ridge mantle may eventually transit^46^ beneath continental rifts, uplifting its eastern shoulder. In this view, no heat anomalies are required to explain the high topography of the eastern margins of both the Red Sea and Dead Sea faults.

**Figure 7:**
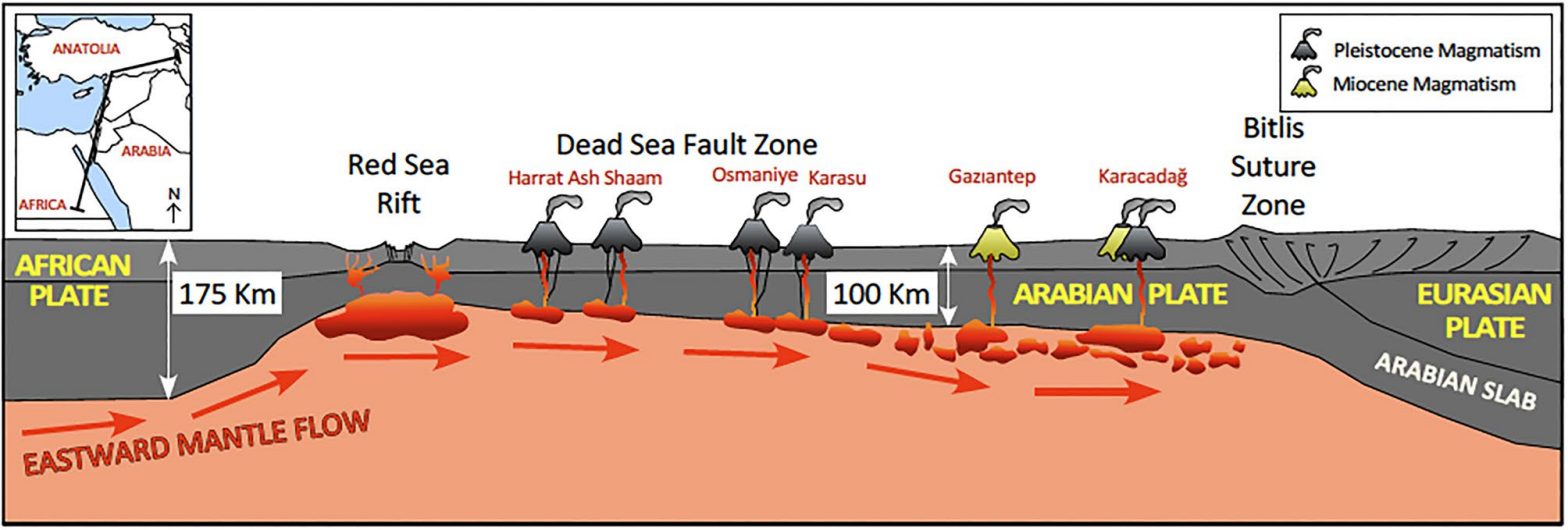
Schematic cross-section cartoon of the region. Major tectonic features and lithosphere/asthenosphere boundary are shown from the Red Sea Rift and the Dead Sea Fault Zone, throughout the Arabian foreland and until the Eurasian-Arabian convergent zone. Hot, buoyant asthenospheric mantle migrates from the rift region towards to the east, locally acting as a source for alkali basaltic volcanism. Insert shows trace of section.

Very long-lasting (20 Ma) homogeneous magmatism produced a large amount of alkali basalts on the western portion of the Arabian Plate, close to the Africa-Arabia margin, from the Oligocene to the Pleistocene and from the southwest (i.e., Yemen) to the northwest (south-eastern Turkey). The occurrence and persistence of an asthenospheric mantle source under the study area is interpreted in the framework of the dynamics between the Arabian and African Plates, which led to the formation of the Dead Sea Fault Zone to the north, and the Red Sea Rift to the south. The divergence between these two plates is still in an embryonic stage, and close to the study area the margin is a strike-slip plate boundary, with a minimal extensional component. Beneath the Red Sea Rift (and in its northern termination represented by the DSFZ), a hot, buoyant asthenospheric mantle reaches the rift zone and partially melts (Fig. 7). Since the Red Sea Rift was and still is in an immature stage, the upwelling asthenosphere does not produce large amounts of basaltic melts in the axial zone. This effect is even stronger under the DSFZ, which is a transcurrent rather than a passive margin. While some magmas come to the surface at the plate margin, portions of still hot, fertile asthenosphere continue their eastward migration and are then stored beneath the western portion of Arabian lithosphere, which is much thinner (100 km)^47^ than the African one (up to 175 km)^48^. Given the large difference in thickness between the two plates, the eastward migration of the asthenosphere implies its upwelling, creating favourable conditions for further partial melting induced by decompression. As highlighted by analogue modelling, magmas are frequently stored not only in the axial zone of rifts, but tend to migrate towards the rift shoulders^49^. The geochemical imprint of these magmas (e.g. REE distribution), as well as pressure estimates reveal that they formed in the mantle asthenosphere in the garnet stability zone (≥ 60 km depth), but at depth lower than 100 km. Neither rift processes nor extensional tectonics have developed in the study area, but local tensional and transtensional activity is responsible for discrete events of restricted asthenosphere upwelling and its decompression melting. This mechanism has also provided some pathways for the ascent of the magmas, generating scattered activity in the Gaziantep Basin and a wide, persistent (> 11 Ma) magma chamber under the Karacadağ shield volcano. More recently, the development of transform fault zones at the north-eastern margin of the Arabian Plate, here represented by the northern termination of the DSFZ, favoured upwelling of the asthenospheric mantle and the formation of Na-alkaline magmas in the Osmaniye and Karasu volcanic fields during the Pleistocene. During its north-eastward migration, the upper asthenosphere, i.e. the low-velocity layer beneath the Arabian Plate, may have retained larger amounts of partial melt, which remained stagnant at the slab flexure, where the north-eastern Arabian Plate subducts beneath Eurasia.

## Methods

### Major and trace elements

Whole rock major and trace element contents (Table 1 and Supplementary Table 1) were determined at the Dipartimento di Scienze della Terra of the Università di Pisa by X-Ray Fluorescence (XRF) and Inductively Coupled Plasma-Mass Spectrometry (ICP-MS) methods using an ARL 9400 XP^+^ and a Fisons PQ2 Plus instrument, respectively. FeO content was measured via titration. Loss on Ignition was determined by gravimetry at 1000 °C after pre-heating at 110 °C.

### Radiogenic isotopes

Sr–Nd-Pb radiogenic isotope analyses were performed at the Istituto di Geoscienze e Georisorse of the Italian National Research Council (IGG-CNR) in Pisa (Italy). Lead was extracted by matrix after eluting with HBr and HCl with AG50W-X8 200–400 mesh anionic resin, whereas Sr and REE were collected in HCl solution through ion-exchange chromatography columns equipped with AG1-X8 100–200 mesh cationic resin and subsequently Nd was extracted from REE eluates, in 0.26 N HCl solution through an Eichrom Ln resin. Sr, Nd and Pb separates were loaded onto Re filaments (99.98% for Sr and Nd, and 99.999% “zone refined” for Pb) and measured with Finnigan Mat 262 thermal ionization mass spectrometer. Strontium and Nd isotopic compositions were acquired in dynamic mode, are results were corrected for mass fractionation using ^86^Sr/^88^Sr = 0.1194 and ^146^Nd/^144^Nd = 0.7219. The Sr standard NIST SRM 987 and the Nd standard J-Ndi1 yielded average values of ^87^Sr/^86^Sr = 0.710239 ± 0.000017 (2SD; n = 43) and ^143^Nd/^144^Nd = 0.512106 ± 0.000012 (2SD; n = 16), respectively; measured values were adjusted at 0.710250 for ^87^Sr/^86^Sr, whereas no Nd isotope adjustment was required. The Sr and Nd blanks were respectively about 2.3 ng and 1.0, which are negligible for the analysed samples. Lead isotope analyses were measured in static mode. Replicate analyses of Pb isotope ratios are accurate within 0.25‰ (2SD) per mass unit, after applying mass discrimination corrections of 0.15 ± 0.01% per mass unit relative to the NIST SRM 981 reference composition^50^^.^ Pb blanks were of the order of 0.2–0.4 ng, and no blank correction was made. Full results and age correction are presented in Supplementary Table 2.

### Boron isotopes

Ten (10) representative samples were selected for B contents and B isotope ratio determinations. Analyses were performed at the Istituto di Geoscienze e Georisorse of the Italian National Research Council (IGG-CNR) in Pisa (Italy) via Multi-Collector ICP-MS technique after B extraction from matrix, as described into details here below. Approximately 0.2–0.5 g of powder rocks samples were merged in Pt-Ir (95–5%) crucibles with K_2_CO_3_, with K_2_CO_3_/rock ratio > 4. K_2_CO_3_ is used because of its high solubility facilitates rapid aqueous leaching of the resulting fusion cake. Sample + K_2_CO_3_ mixture are then fused at temperature of ~ 1000 °C. After cooling, boron was then extracted into solution via overnight immersion in high pH B-free water. Then the water solution, along with the insoluble phases were transferred in polypropylene tubes, and, after shaking, water solution containing B was separated by solid residue by centrifuging. Boron was then extracted from the solution with a three-step chemical separation procedure, very close to the method described by Tonarini et al.^51^: the first step of sample purification was performed using boron-specific ion-exchange resin 20–50 mesh amberlite, loading approximately 0.3–0.4 ml of resin in Savillex PFE Teflon micro-columns, previously cleaned with 1.5 N HCl and conditioned with Ultrapure high-pH water and ammonia. Sample was loaded at pH > 10 and rinsed with ultrapure high-pH water and ammonia, then boron was eluted with 1.5 HCl N, and collected in concave bottom Savillex PFE teflon beakers and dried overnight. To avoid boron-loss during this step, mannitol was also added to the eluted solution, and hot plate were at T < 70 °C. The day after, samples were dissolved in very weak (0.015 N) HCl and then passed through AG 50 W-X8 (200–400 mesh) cation-exchange resin. B was immediately collected with 0.015 N HCl, then the solution was adjusted to pH > 10 adding 0.8 ml of NH_4_OH (1.5 N), and a third purification step was performed, again passing the sample into the 20–50 mesh amberlite resin, following the same procedure of the first day, just modified by collecting B from amberlite in the last step with 2% HNO_3_, to have a solution ready to be measured via ICP-MS, after eventual dilution. All the chemical purification steps were performed in a class 1000 clean room lab, using B-free ultrapure reagents. Ultrapure water was obtained starting from Milli-Q water (resistivity of 18.2 MΩ cm^−1^), and subsequent sub-boiled distillation using Savillex DST-1000; ultrapure HCl was obtained starting from azeotropic solution of Iso-pro analysis HCl and two subsequent steps of sub-boiled distillation using Savillex DST-1000; ultrapure HNO_3_ was obtained starting from Suprapur HNO_3_ and two subsequent steps of sub-boiled distillation using Savillex DST-1000; ultrapure B-free NH_4_OH was obtained with sub-boiled distillation of Ultrapure NH_4_OH, adding mannitol to the starting solution to prevent B volatilization.

The purified solutions were measured on a Thermo Neptune Plus MC-ICP-MS at CNR-IGG Pisa, specially tuned for B isotope analysis and maximum ^11^B/^10^B stability. After B concentration measurements, all the samples were diluted to a B concentration of ≈ 25 ng/g, and bracketed with 25 ng/g solution of NBS 951 boric acid standard. The analytical procedure for B isotope analysis consisted of sample-reference bracketing, using NIST SRM 951, and an on-peak zero blank correction^52^. A triplicate of analysis was done for each sample during same analytical session. The result is given as average delta value without further normalisation. Because of the large mass fractionation during MC-ICP-MS analyses of boron, the instrument was tuned before each analytical session for maximum stability rather than maximum intensity adjusting the sample gas flow following the procedure suggested by Foster (2008)^53^. A wash time of 250 s after each sample and reference analysis in order to overcome the well-known wash-out problem of boron^54^. This allowed to have a blank signal always < 0.5% (≈1 mV on ^11^B peak for the blank, against ≈180–250 mV ^11^B peak for the samples and the standards. The given delta notation (δ^11^B) represent the ‰ deviation from the NIST SRM 951 standard, with a certified ^11^B/^10^B ratio of 4.04362^55^.

Within run errors on individual runs (n = 3) were in the order of 0.1 ± 0.2 ‰. Several samples and in-house standards (incl. Mt. Etna IAEA standard B5) were re-processed and re-produced the original values^56^ to within 0.4 ‰ or better. The accuracy of the measurement was monitored by: 33 replicate analyses of shelf NBS 951 gave an average δ^11^B of − 0.05 ± 0.28 (2σ), 15 replicate analyses of NBS 951 after full chemistry gave an average δ^11^B of − 0.42 ± 0.56 (2σ), 7 replicate analyses of the IAEA standard B1 (seawater) which gave an average δ^11^B of + 39.38 ± 0.27 (~ + 39)^57^, and 3 replicate analyses of the JB2 (basalt) which gave an average δ^11^B of + 7.25 ± 0.57 (+ 7.33 ± 0.37 (2σ)^57^, 2 replicate analyses of IAEA B-5 standard gave − 4.49 ± 0.20 and − 4.20 ± 0.52 (− 3.95 ± 0.32)^57^; three replicates of in-house BN boric acid standard gave an average of − 13.33 ± 0.40 (TIMS average − 13.59 ± 0.40).

The boron concentrations were measured alongside δ^11^B ratios at IGG-CNR-Pisa. Known concentrations of 951 boron solution and internal 50 ng/g and 10 ng/g standard solutions were used to construct a calibration line, which was used to determine the unknown sample boron concentrations using the known volumes of reagents used during sample extraction and purification. The uncertainties of these measurements were in the order of 10%.

## Supplementary Information

Supplementary Information 1.

Supplementary Information 2.
