# Supplementary material for: A heterogeneous subcontinental mantle under the African–Arabian Plate boundary revealed by boron and radiogenic isotopes
Source: Sci Rep. 2021 May 27;11:11230. doi: 10.1038/s41598-021-90275-7 (PMC8160130; doi:10.1038/s41598-021-90275-7)
Supplement: Supplementary file 1 — Supplementary Information 1. [file 41598_2021_90275_MOESM1_ESM.docx]

**SUPPLEMENTARY MATERIAL**

**A heterogeneous subcontinental mantle under the African-Arabian plate boundary revealed by boron and radiogenic isotopes**

Samuele Agostini^1^, Paolo Di Giuseppe^1^, Piero Manetti^2^, Carlo Doglioni^3,4^, Sandro Conticelli^2,5^

1. *Istituto di Geoscienze e Georisorse, Sede principale di Pisa, Consiglio Nazionale delle Ricerche, Via Moruzzi, 1, I-56124, Pisa, Italy*
2. *Dipartimento di Scienze della Terra, Università degli Studi di Firenze, Via Giorgio La Pira, 4, I-50121, Firenze, Italy*

*3) Dipartimento di Scienze della Terra, Università La Sapienza, Roma, Piazzale Aldo Moro 5, I-00185 Roma, Italy*

*4) Istituto Nazionale di Geofisica e Vulcanologia, via Vigna Murata 605, I-00143 Roma, Italy*

*5) Istituto di Geologia Ambientale e Geoingegneria, Sede principale di Montelibretti, Consiglio Nazionale delle Ricerche, Area della Ricerca di Roma1 - Montelibretti, Via Salaria Km 29,300, I-00015 Monterotondo (RM), Italy.*

Corresponding author: Samuele Agostini, s.agostini@igg.cnr.it

**Estimate of melt segregation temperature and pressure**

The temperature and pressure at which magmas segregated from their peridotitic source were estimated using primary melt compositions (e.g., Albaréde, 1992; Herzberg et al., 2007; Putirka et al., 2007; Lee et al., 2009; Plank & Forsyth, 2016). Calculation and results are in Supplementary Table **3**). Starting from major element abundances, we calculated the primary magma compositions adding back equilibrium olivine until magmas equilibrates with olivine Fo_90_, using the equations by Pearce (1978), and calculating the composition of equilibrium olivine using K_D_ (Fe/Mg)^ol/liq^ = 0.31 (Putirka, 2005). As evidenced in Supplementary Table 3, major element composition of samples was normalized at 100% on anhydrous base, assuming an original Fe_2_O_3_/FeO ratio of 0.15 as from Middlemost (1989). According to our model, the amount of olivine added to equilibrate starting melt compositions to Fo_90_ source was estimated in between 16 and 25% for the selected samples. Then, to calculate magma segregation temperatures and pressures, we used the thermobarometer from Lee et al. (2009), as modified by Plank & Forsyth (2016). Here, the H_2_O content in the melts was estimated by fractionation correction of Ce (ppm), assuming that the magmas emplaced in the study areas have the same H_2_O/Ce ratios as oceanic basalts (~ 200; Herzberg et al., 2007). The estimated H_2_O content was in the range of 0.8 to 2.3 wt.%.

Results show that melting temperatures (T °C) varies from 1365 to 1502 °C, whereas estimated pressure (P) varies from 1.17 to 3.06 GPa, that is ≈36-93 km of depth (Supplementary Table **3**). Model results are also plotted in Figure 6**.** In the main text, as well as in Fig. 6, we excluded samples potentially affected by significant amount of crustal contamination, highlighted in grey in Supplementary Table 3 (see discussion section of main text for full details). Hence, there we reported narrower intervals of 2.03-3.06 GPa and 1428-1501 °C for pressure and temperature, respectively.

Recently, different authors made similar estimate on basaltic rocks from the same region. Our data are in good agreement with those of Reid et al. (2017), whose estimates range from 1.80 to 2.96 GPa (pressure) from 1394 to 1426 (T_P_) for different basalts from Eastern and Southeastern Anatolia and with those of Nikogosian et al. (2018), estimating Tp ≈1530-1455 °C and P≈2.7-3.3 GPa for Karacadag and T_P_ ≈1430-1355 °C and P≈1.9-2.8 GPa for Ceyhan-Osmaniye. Calculations in McNab et al. (2018) span over a much wider interval, with T_P_=1360-1490 °C and P = 1.5-3.5 GPa: here, sample with higher values are in agreement with our data, and sample with lower values, e.g. T_P_<1400 °C and P < 2.5 are much more similar to the model we performed with more evolved sample, clearly retaining some imprint of crustal assimilation. Similar estimates for Osmaniye area are also reported in Oyan (2018), with a range of 2.8-3.5 GPa and 1275-1320 °C for basaltic samples, and ≈3.7 GPa and 1360-1390 °C for basanitic rocks: this data are in some agreement with our model for pressure estimates, whereas temperature are much lower than any other reported calculations. No details on these calculations are given in Oyan (2018), in the main text or in the supplementary materials, it is only stated that model used is that from Lee et al. (2009), and it is not clearly stated if they deal with temperature or potential temperature, then these data can not easily be compared with our data as well as the other above reported.

**Cited References in Supplementary Materials**

Albaréde, F. How Deep Do Common Basaltic Magmas Form and Differentiate? *J. Geophys. Res*. **97**, 10.997-11.009 (1992).

Herzberg, C. *et al.* Temperatures in ambient mantle and plumes: constraints from basalts, picrites and komatiites. *Geochem. Geophys. Geosyst* **8**, 1-34 (2007).

Lee, C-T., A., Luffi, P., Plank, T., Dalton, H. & Leeman, W. Constraints on the depths and temperatures of basaltic magma generation on Earth and other terrestrial planets using new thermobarometers for mafic magmas. *Earth Planet. Sci. Lett*. **279**, 20-33 (2009).

McNab, F., Ball, P.W., Hoggard, M.J. & White, N.J. Neogene Uplift and Magmatism of Anatolia: Insights From Drainage Analysis and Basaltic Geochemistry. *Geochem. Geophys. Geosyst*. **19**, 175–213 (2018).

Middlemost, E. Iron oxidation ratios, norms and the classification of volcanic rocks. *Chem. Geol.* **77**, 19-26 (1989).

Nikogosian, I. K., Bracco Gartner, A. J. J.,van Bergen, M. J., Mason, P. R. D., & van Hinsbergen, D. J. J. Mantle sources of recent Anatolian intraplate magmatism: A regional plume or local tectonic origin?. *Tectonics*, **37**, 4535-4566 (2018).

Oyan, V. Petrogenesis of the Quaternary mafic alkaline volcanism along the African-Anatolian plates boundary in Turunçlu-Delihalil (Osmaniye) region in southern Turkey. *Lithos* **314-315**, 630-645 (2018).

Plank, T. & Forsyth, D.W. Thermal structure and melting conditions in the mantle beneath the Basin and Range province from seismology and petrology. *Geochem. Geophys. Geosyst.* **17**, 1312-1338 (2016).

Pearce, T.H. Olivine fractionation equations for basaltic and ultrabasic liquids. *Nature* **276**, 771-774 (1978).

Putirka, K. D. Mantle potential temperatures at Hawaii, Iceland, and the mid-ocean ridge system, as inferred from olivine phenocrysts: evidence for thermally driven mantle plume. *Geochem. Geophys. Geosyst* **6**, doi:10.1029/2005GC000915 (2005).

Putirka, K.D., Perfit, M., Ryerson, F.J. & Jackson, M.G. Ambient and excess mantle temperatures, olivine thermometry, and active vs. passive upwelling. *Chem. Geol.* **241**, 177–206 (2007).

Reid, M., Schleiffarth, W.K., Cosca, M.A., Delph, J.R., Blichert-Toft, J. & Cooper, K.M. Shallow melting of MORB-like mantle under hot continental lithosphere, Central Anatolia. *Geochem. Geophys. Geosyst.* **18**, 1866-1888 (2017).

***Supplementary Figure 1***

***
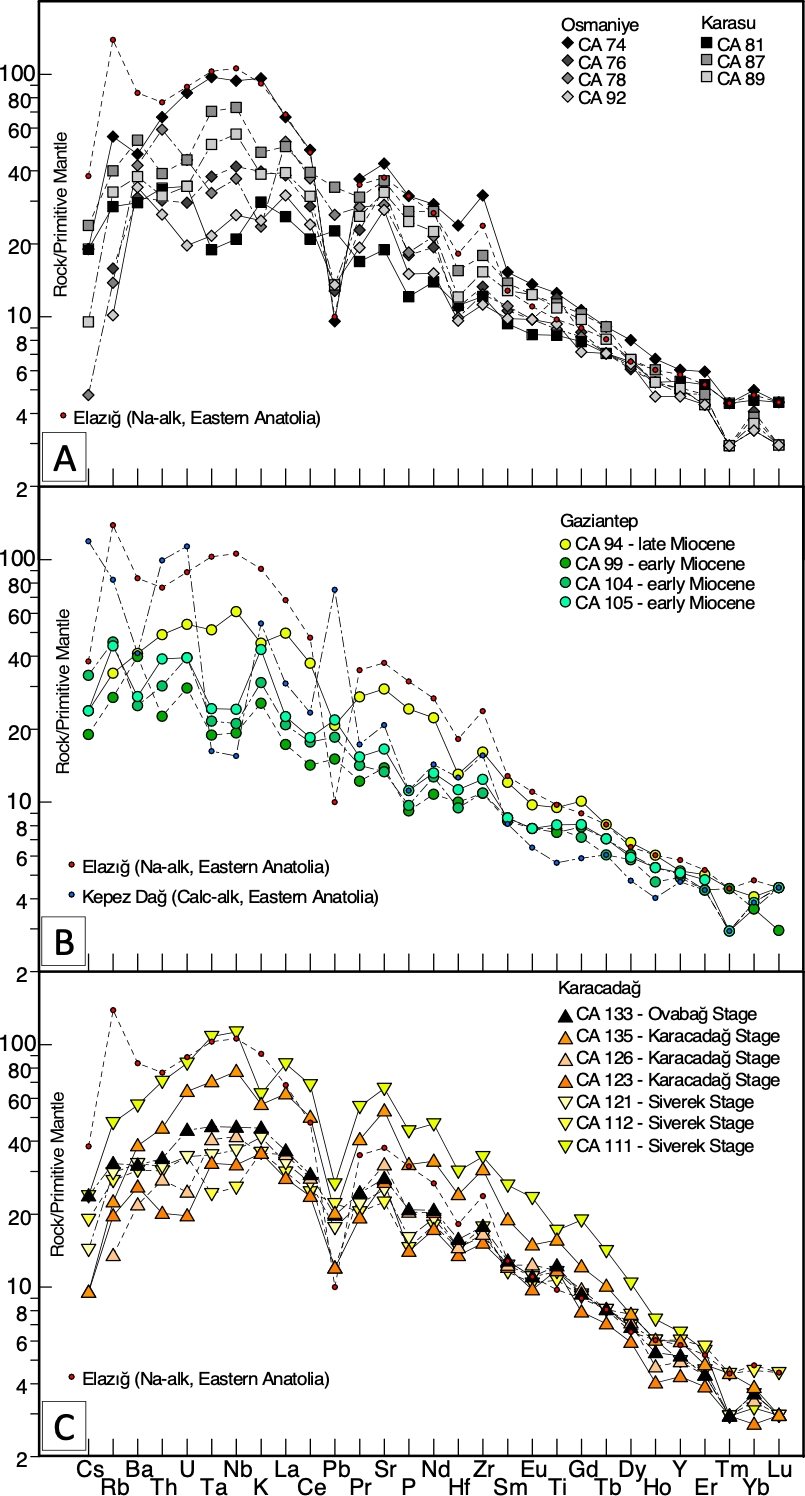
***

***Supplementary Figure 1. Primitive Mantle-normalised diagrams for all of the studies samples.*** ***A****, Dead Sea Fault Zone;* ***B,*** *Gaziantep;* ***C****, Karacadağ. A Na-alkaline basalt of Elazığ (Eastern Anatolia, from Di Giuseppe et al., 2017) and a subduction-related basaltic andesite of Kepez Dağ volcanic complex (Central-Eastern Anatolia, from Di Giuseppe et al., 2019) are reported for comparative purposes. Normalisation factors from McDonough and Sun (1995).*

***Supplementary Table 1.*** *Major Elements, CIPW Norm and Trace elements of collected samples*

(see also attached excel file)

***Supplementary Table 2.*** *Measured and age-corrected data for Sr-Nd-Pb radiogenic isotopes.*

(see also attached excel file)

******

***Supplementary Table 3.*** *Estimates of Temperature, Pressure and depth of magma segregation*

(see also attached excel file)

**
